# A sense of direction

**DOI:** 10.7554/eLife.110796

**Published:** 2026-03-02

**Authors:** Aleksandr Pakhomov, Dmitry Kishkinev

**Affiliations:** 1 School of Life Sciences, Keele University Keele United Kingdom

**Keywords:** Spodoptera frugiperda, insect migration, nocturnal navigation, geomagnetic cues, Other, Fall armyworm

## Abstract

Migratory moths use both magnetic and visual cues for navigation when travelling long distances in the dark.

**Related research article** Ma YB, Wan GJ, Ji Y, Chen H, Gao BY, Yu DH, Warrant EJ, Wu Y, Chapman JW, Hu G. 2025. Geomagnetic and visual cues guide seasonal migratory orientation in the nocturnal fall armyworm, the world’s most invasive insect. *eLife* **14**:RP109098. doi: 10.7554/eLife.109098.

Every year billions of migratory insects travel for thousands of kilometres across continents. Among them are moths, which have a number of important ecological roles: they pollinate plants, they move nutrients between regions, and they serve as seasonal food for birds and other animals. To complete their journeys, these insects must maintain a steady flight direction while moving through changing and often unfamiliar environments.

Daytime migrants such as butterflies and hoverflies can use the sun for navigation: by tracking the sun’s position across the sky, and compensating for its daily movement, these insects can maintain consistent headings over long distances (Mouritsen and Frost, 2002; Massy et al., 2021). However, how do moths and other insects that travel at night navigate when the sun is nowhere to be seen? Visual information is often limited to nearby landmarks and although the stars could, in principle, provide guidance, the night sky is frequently obscured by clouds. Moreover, moth migration often occurs at high altitudes (Chapman et al., 2010), where familiar features on the ground may not be visible. Navigating over long distances under these conditions requires directional cues that are both accurate and consistently available. What cues do moths use to navigate as they migrate thousands of kilometres in the dark?

For decades, scientists have proposed that nocturnal insects might use the Earth’s magnetic field for guidance (Baker and Mather, 1982), similar to migratory birds, because the geomagnetic field is continuously available and provides global directional information. It has also been suggested that moths could use patterns of stars or the Milky Way (Foster et al., 2018). For many years there was little evidence to support these ideas, but that changed when researchers discovered that the Australian Bogong moth (*Agrotis infusa*) – a species that migrates between south east Australia and the Australian Alps – uses both the Earth’s magnetic field and the stars to navigate (Dreyer et al., 2018; Dreyer et al., 2025).

Importantly, it seems that Bogong moths cannot navigate using the Earth’s magnetic field on its own – they also require some sort of visual cue. In the wild there are multiple visual cues, including stars and various landmarks; in lab-based experiments, the visual cue is often a black triangle that simulates a mountain. Do other migratory moths use the Earth’s magnetic field in combination with visual landmarks in a similar way? Now, in eLife, Gao Hu (Nanjing Agricultural University and Guiyang University) and co-workers – including Yi-Bo Ma and Gui-Jun Wan as joint first authors – report that the answer to this question is yes (Ma et al., 2025). The researchers – who are based at institutions in China, Sweden and the United Kingdom – performed experiments on a type of moth called the fall armyworm (*Spodoptera frugiperda*), a highly invasive pest species that spreads across large regions and damages important crops.

Ma et al. asked a simple question: can these moths navigate using just the Earth’s magnetic field, or do they need additional sensory information? To address this, the researchers used a flight simulator system that allowed individual moths to fly in any direction while attached to a tether (Dreyer et al., 2021). The researchers were able to control the magnetic field experienced by the moth, and could also add visual landmarks (usually black triangles). This allowed them to monitor the impact of the magnetic field and the visual landmarks on the direction of flight.

When the magnetic field and the visual landmark were aligned, the moths flew in a specific direction that matched the direction taken by moths in the wild (Figure 1). This showed that fall armyworms can use magnetic information to navigate, at least when they are provided with a visual landmark. However, they lost this ability when the landmark was removed or when the tests were conducted in near darkness. This suggests that the magnetic compass in these moths depends on visual context to function reliably.

**Figure 1.**
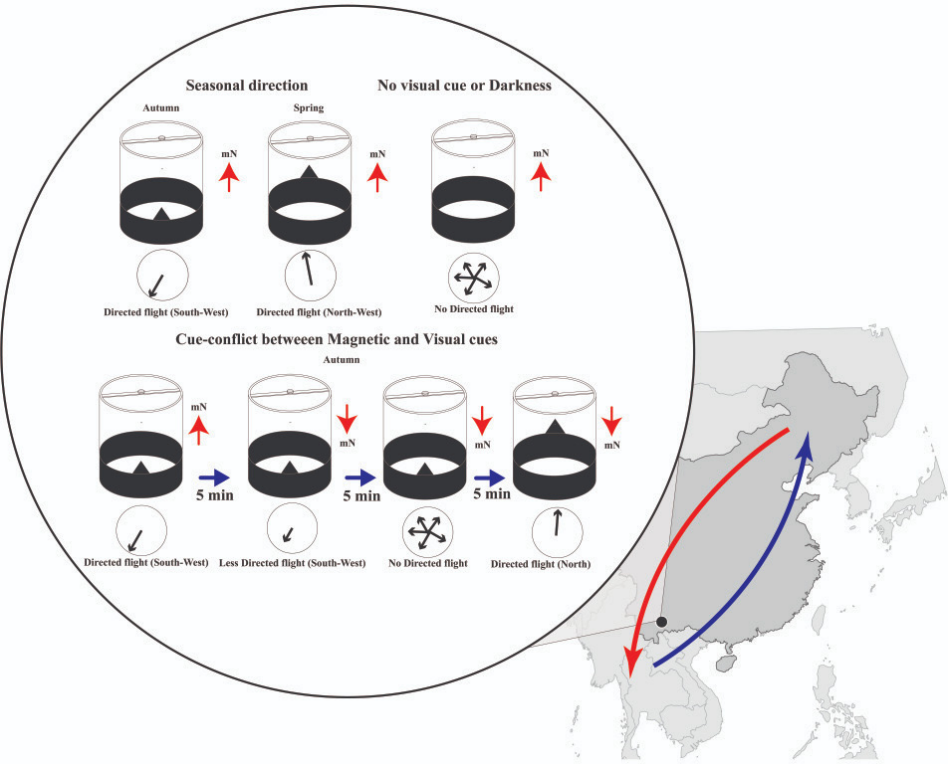
How do fall armyworm moths navigate long distances in the dark? Ma et al. used a flight simulator (cylindrical structure) to study the direction of flight of a moth (not shown) under controlled magnetic and visual conditions. When the magnetic field points towards magnetic North (mN; red arrow), and the visual cue (black triangle) is to the geographic South (upper left), the moths fly in a south-westerly direction, which is the direction they take in autumn. When the magnetic field points towards magnetic North, and the visual cue is to the geographic North (upper middle), the moths fly in a north-westerly direction, which is the direction they take in spring. However, when the visual cue is removed or the experiments are conducted in darkness (upper right), the moths fly in a variety of directions. To explore what happens when there is a conflict between the magnetic and visual signals, Ma et al. started with the autumn set-up: magnetic field pointing towards magnetic North, and the visual cue to the geographic South (lower left). As before the moths fly in a south-westerly direction. If the direction of the magnetic field is reversed (lower, second from left), the moths continue to fly in a south-westerly direction. However, after five minutes (lower, second from right), the moths start to fly in a variety of directions. If the visual cue is then moved to the geographic North to remove the conflict between the magnetic and visual signals (lower right), the moths start to fly towards the geographic North, which is towards the magnetic South in this configuration, which is the appropriate direction for autumn migration. Ma et al. also did similar experiments in spring with similar outcomes (not shown here). Fall armyworm populations breed year-round in tropical southeast Asia and south China (the black dot indicates the study site in Yunnan Province). In spring, populations migrate northward into northern and northeastern China (blue arrow) to breed. The offspring produced during the summer migrate southward in autumn (red arrow).

The researchers also created situations in which the magnetic and visual cues conflicted (for example, the magnetic field direction was reversed while the visual landmark remained in the same position, or vice versa). In these trials, the moths initially followed the visual landmark rather than the magnetic cue. After a few minutes, however, direction was lost, but it was restored when the magnetic and visual cues were made consistent with each other.

Together, these findings challenge the idea that these moths use magnetic orientation as a simple, stand-alone compass. Instead, it seems that they integrate magnetic and visual signals to choose their heading (and presumably prioritise visual signals when conflicts arise). Visual landmarks may help calibrate the magnetic compass, or provide a spatial framework that enables the animal to interpret magnetic information. Without such contextual information, magnetic cues alone are not enough to allow the moths to navigate.

An important open question is how this system operates under more naturalistic conditions, where moths are exposed to multiple visual landmarks of varying salience, rather than just one landmark. In particular, how is information from all possible cues (landmarks, the magnetic field, stars etc) integrated, and how does the insect brain process such complex information?

## References

[bib1] Baker RR, Mather JG (1982). Magnetic compass sense in the large yellow underwing moth, *Noctua pronuba* L. Animal Behaviour.

[bib2] Chapman JW, Nesbit RL, Burgin LE, Reynolds DR, Smith AD, Middleton DR, Hill JK (2010). Flight orientation behaviors promote optimal migration trajectories in high-flying insects. Science.

[bib3] Dreyer D, Frost B, Mouritsen H, Günther A, Green K, Whitehouse M, Johnsen S, Heinze S, Warrant E (2018). The earth’s magnetic field and visual landmarks steer migratory flight behavior in the nocturnal Australian Bogong moth. Current Biology.

[bib4] Dreyer D, Frost B, Mouritsen H, Lefèvre A, Menz M, Warrant E (2021). A guide for using flight simulators to study the sensory basis of long-distance migration in insects. Frontiers in Behavioral Neuroscience.

[bib5] Dreyer D, Adden A, Chen H, Frost B, Mouritsen H, Xu J, Green K, Whitehouse M, Chahl J, Wallace J, Hu G, Foster J, Heinze S, Warrant E (2025). Bogong moths use a stellar compass for long-distance navigation at night. Nature.

[bib6] Foster JJ, Smolka J, Nilsson DE, Dacke M (2018). How animals follow the stars. Proc Roy Soc B. Biological Sciences.

[bib7] Ma YB, Wan GJ, Ji Y, Chen H, Gao BY, Yu DH, Warrant EJ, Wu Y, Chapman JW, Hu G (2025). Geomagnetic and visual cues guide seasonal migratory orientation in the nocturnal fall armyworm, the world’s most invasive insect. eLife.

[bib8] Massy R, Hawkes WLS, Doyle T, Troscianko J, Menz MHM, Roberts NW, Chapman JW, Wotton KR (2021). Hoverflies use a time-compensated sun compass to orientate during autumn migration. Proc Roy Soc B. Biological Sciences.

[bib9] Mouritsen H, Frost BJ (2002). Virtual migration in tethered flying monarch butterflies reveals their orientation mechanisms. PNAS.

